# The Whys and Wherefores of Transitivity in Plants

**DOI:** 10.3389/fpls.2020.579376

**Published:** 2020-08-31

**Authors:** Felipe F. de Felippes, Peter M. Waterhouse

**Affiliations:** Centre for Agriculture and the Bioeconomy, Institute for Future Environments, Queensland University of Technology (QUT), Brisbane, QLD, Australia

**Keywords:** transitivity, small RNAs, post-transcriptional gene silencing, gene silencing, phasiRNAs, tasiRNAs, siRNAs, RDR6

## Abstract

Transitivity in plants is a mechanism that produces secondary small interfering RNAs (siRNAs) from a transcript targeted by primary small RNAs (sRNAs). It expands the silencing signal to additional sequences of the transcript. The process requires RNA-dependent RNA polymerases (RDRs), which convert single-stranded RNA targets into a double-stranded (ds) RNA, the precursor of siRNAs and is critical for effective and amplified responses to virus infection. It is also important for the production of endogenous secondary siRNAs, such as phased siRNAs (phasiRNAs), which regulate several genes involved in development and adaptation. Transitivity on endogenous transcripts is very specific, utilizing special primary sRNAs, such as miRNAs with unique features, and particular ARGONAUTEs. In contrast, transitivity on transgene and virus (exogenous) transcripts is more generic. This dichotomy of responses implies the existence of a mechanism that differentiates self from non-self targets. In this work, we examine the possible mechanistic process behind the dichotomy and the intriguing counter-intuitive directionality of transitive sequence-spread in plants.

## Introduction

The proper regulation of gene expression is essential for the development and the adaptation of plants to their environment. Gene silencing mediated by small RNAs (sRNAs) plays a major role in this process, leading to the downregulation of gene activity at the transcriptional (transcriptional gene silencing [TGS]) or post-transcriptional level (post-transcriptional gene silencing [PTGS]) (Bologna and Voinnet, 2014; Li et al., 2017). In addition, sRNAs form one of the main lines of defense against virus infection and also contributes to controlling the spread of opportunistic sequences, such as transposons (Li et al., 2017). There are two modes of sRNA biogenesis, as microRNAs (miRNAs) and as small interfering RNAs (siRNAs). The sRNAs are processed from precursor double-stranded RNAs (dsRNAs) into 20 to 24 nt molecules by DICER-LIKE enzymes (DCLs). These sRNAs are then loaded into an ARGONAUTE (AGO) to form the RNA-induced silencing complex (RISC), the effector complex of the RNA silencing pathway, resulting in DNA methylation, transcript cleavage, or translation inhibition of its targets in a sequence specific fashion (Bologna and Voinnet, 2014).

An interesting aspect of sRNA-mediated silencing is that it can be amplified. In some species, transcript targeting by sRNAs can lead to the production of a second wave of sRNAs, known as secondary siRNAs (Sijen et al., 2001; Vaistij et al., 2002; Nicolás et al., 2003). In plants, the generation of these molecules involves RNA-dependent RNA polymerase 6 (RDR6), which uses the target single-stranded RNA (ssRNA) as a template for the synthesis of a dsRNA molecule that is converted to sRNAs mainly by the hierarchical action of DCL4 and DCL2 (Vaistij et al., 2002; Deleris et al., 2006; Fusaro et al., 2006; Moissiard et al., 2007; Mlotshwa et al., 2008). As a result, not only the abundance of sRNAs is increased, but also the spreading of the silencing signal into areas adjacent to the initial targeted region. This process, most often referred to as transitivity, is of critical importance to many plant processes. Phased siRNAs (phasiRNAs), which include trans-acting siRNAs (tasiRNAs), are a subclass of siRNAs that rely on transitivity for their biogenesis (Deng et al., 2018; de Felippes, 2019). PhasiRNAs play a central role in development and in the response to abiotic and biotic stresses in plants (Deng et al., 2018). Transitivity is also important for the non-cell autonomous function of sRNAs. Besides regulating gene expression in the location where they are produced, siRNAs can also move and drive silencing in neighboring cells (Brosnan and Voinnet, 2011; Liu and Chen, 2018). The distance they can travel from cell-to-cell and *via* the vasculature can be dramatically increased by means of transitivity (Himber et al., 2003; de Felippes et al., 2011; Liang et al., 2012). Transitivity is required not only for the generation of the mobile signal, but also for its perception at the recipient tissue (Klahre et al., 2002; García-Pérez et al., 2004; Brosnan et al., 2007). Transitivity is also involved with the response to virus infection, amplifying the primary pool of viral siRNAs and spreading the antiviral silencing signal systemically to give plant-wide immunization (Willmann et al., 2011).

Despite its biological importance, several aspects of transitivity in plants are still poorly understood. In contrast to organisms supporting transitivity outside of the plant kingdom, the spread of the silencing signal is bi-directional in plants, with secondary sRNAs originating up- and downstream of the target region (Vaistij et al., 2002). Yet, the expansion of the silencing signal in plants shows a clear tendency toward the 3′ region of the transcript (Braunstein et al., 2002; Petersen and Albrechtsen, 2005; Bleys et al., 2006b; Haque et al., 2007; Moissiard et al., 2007; Aregger et al., 2012; Dadami et al., 2014; Wroblewski et al., 2014; Han et al., 2015; de Felippes et al., 2020). Another interesting aspect of transitivity in plants is the apparent susceptibility of transgenes in becoming template of RDR6. While endogene mRNAs targeted by sRNAs originating from synthetic hairpin RNAs (Waterhouse et al., 1998; Wesley et al., 2001) are usually degraded without the production of secondary sRNAs, mRNAs from transgenes often become templates for co-suppression through dsRNA synthesis and secondary siRNA production (Béclin et al., 2002; Vaistij et al., 2002; Himber et al., 2003; Kościańska et al., 2005; Miki et al., 2005; Petersen and Albrechtsen, 2005; Bleys et al., 2006b; Aregger et al., 2012). Thus, plants appear able to differentiate self from non-self genes, using transitivity to destroy invading sequences. Here, we discuss scenarios frequently touted to explain the proneness of transgenes to transitivity and the recent findings that put terminators and the formation of the 3′ end of mRNAs in the center of this defense mechanism. We also offer a model to explain why transitivity in plants seems to spread preferentially toward the 3′ region of the target transcript.

## Factors Triggering Transitivity

### miRNA-Triggered Transitivity

**Figure 1:**
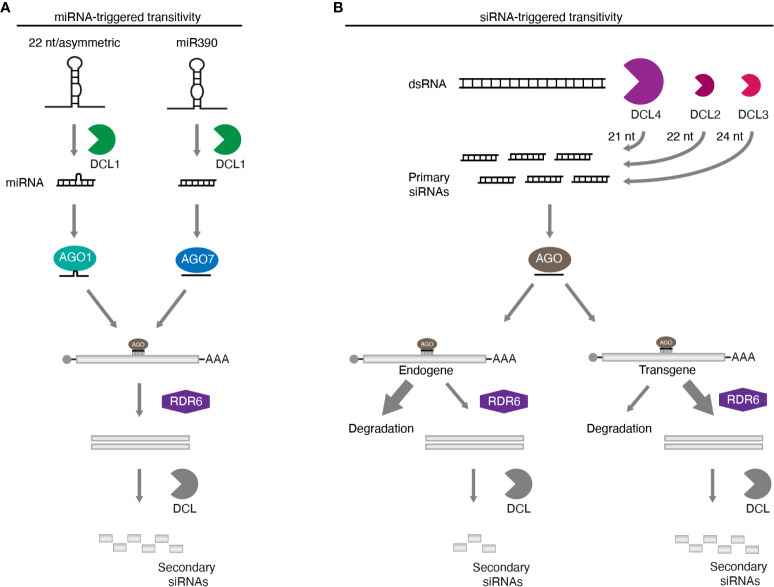
Transitivity mechanisms in plants. **(A)** Transitivity can be initiated by miRNAs that are 22 nt or originate from an asymmetric duplex. Alternatively, secondary siRNA production can also be triggered by the miR390 loaded into AGO7. MiRNA-dependent transitivity is efficiently initiated whether the target is an endogene or not. **(B)** dsRNA precursors are processed into 21, 22, and 24 nt primary siRNAs by the hierarchical activity of DCL4, DCL2, and DCL3, respectively. In contrast to miRNAs, transitivity triggered by siRNAs is less understood and tends to manifest on targets of exogenous origin, such as transgenes, with endogenous sequences being usually resistant to RDR6 routing, as indicated by the proportion of the arrows.

In plants, transitivity can be triggered when transcripts are targeted by miRNAs or siRNAs, including those originating from hairpin RNAs and VIGS. The production of secondary siRNA initiated by miRNAs is a process relatively well understood, and is best exemplified by the biogenesis of tasiRNAs. Most miRNAs are generated as 21 nt molecules, and after loading onto AGOs, direct cleavage followed by degradation of their target mRNA (Bologna and Voinnet, 2014). However, some transcripts such as tasiRNA precursors (*TAS*), serve as templates for RDR6-dependent synthesis of dsRNAs, which are subsequently processed by DCLs (Peragine et al., 2004; Vazquez et al., 2004; Allen et al., 2005; Williams et al., 2005; Yoshikawa et al., 2005). Interestingly, most miRNAs triggering transitivity are 22 nt molecules. This extra nucleotide over the common miRNA size of 21 nt appears to be key to determining, whether or not, the target RNA becomes a template for RDR6-dependent secondary siRNA generation (Chen et al., 2010; Cuperus et al., 2010); and unexpectedly, even a 21 nt miRNA from an asymmetric miRNA/miRNA* duplex (such as a 21 nt miRNA duplexed with a 22 nt complementary miRNA*) can also trigger transitivity (Manavella et al., 2012). The major effector protein for miRNA-guided function (irrespective of the sRNA size) is AGO1 and it is believed that 22 nt long/asymmetric miRNAs can re-program it to foster RDR6 recruitment and secondary siRNA generation (de Felippes, 2019) (**Figure 1A**). A notable exception to this rule is miR390, which initiates tasiRNA biogenesis from *TAS3* (Allen et al., 2005). The 21 nt miRNA, from a 21/21 nt symmetric duplex, would not be expected to initiate transitivity on its target transcript. The *TAS3* mRNA has two miR390 sites, leading to the proposal that the special “two-hit” event can also initiate tasiRNA generation (Axtell et al., 2006). However, it has recently been shown that tasiRNA biogenesis from *TAS3* can also be triggered by a single targeting event mediated by miR390 (de Felippes et al., 2017), indicating that a double-hit is not the mechanism behind miR390’s ability to start transitivity. tasiRNA generation from *TAS3* is also remarkable for being the only *TAS* locus that requires AGO7, not AGO1 (Adenot et al., 2006; Fahlgren et al., 2006; Hunter et al., 2006; Montgomery et al., 2008). Interestingly, miR390 mainly interacts with AGO7, and when this miRNA is loaded into another AGO, such as AGO1 or AGO2, transitivity is abolished (Montgomery et al., 2008). Thus, unlike AGO1 that needs to be programmed by 22 nt/asymmetric miRNAs to trigger transitivity, AGO7 seems to be in a constant state of activation allowing for RDR6 recruitment on the target transcript despite the smaller size of miR390 (**Figure 1A**). Further details on miRNA-triggered transitivity and phasi/tasiRNA biogenesis can be found in the following recent reviews (Deng et al., 2018; de Felippes, 2019).

### siRNA-Triggered Transitivity

Compared to miRNAs, transitivity initiated by siRNAs is less understood. The precursor dsRNA is primarily processed by the hierarchical activity of DCL4, DCL2 and DCL3 into 21, 22 and 24 nt molecules, respectively. DCL4 is the primary processor of dsRNA of different origins, being dominant over DCL2. Only when the former is absent, can DCL2-dependent siRNAs be easily detected (Gasciolli et al., 2005; Xie et al., 2005; Deleris et al., 2006; Fusaro et al., 2006). These 22 nt siRNAs seem to be able to initiate transitivity. Parent et al. (Parent et al., 2014) showed that DCL2 activity promotes secondary siRNA production, corroborating an earlier observation that transitivity requires the action of this enzyme, but not DCL4 (Mlotshwa et al., 2008). These results suggest that, as with miRNAs, siRNA-triggered transitivity relies mainly on the re-programing of AGOs with 22 nt sRNAs (**Figure 1B**). However, two pieces of evidence sit uncomfortably with this scenario. DCL4 is dominant over DCL2, thus much lower levels of 22 nt than 21 nt siRNAs are produced in the normal situation of both DCLs being present (Gasciolli et al., 2005; Xie et al., 2005; Deleris et al., 2006; Fusaro et al., 2006; Moissiard et al., 2007); and secondly, in contrast to miRNAs, siRNA-triggered transitivity is very limited when the target mRNA is from an endogene compared to the situation with transgenic transcripts or viral RNA (**Figure 1B**) (Vaistij et al., 2002; Himber et al., 2003; Kościańska et al., 2005; Miki et al., 2005; Petersen and Albrechtsen, 2005; Bleys et al., 2006b; Aregger et al., 2012). This suggests the existence of an additional mechanism that drives transgenic and viral RNA into the RDR6 pathway.

As for transitivity, transgenes are also more prone than endogenes to become self-silenced. This observed susceptibility of transgenic sequences to silencing (Napoli et al., 1990; van der Krol et al., 1990) led to the development of co-suppression technologies to downregulate gene expression, even before the discovery of siRNAs. In this mechanism, also known as sense-PTGS (S-PTGS), dsRNA molecules are generated from sequences expressed in sense by RDR6-mediated activity, leading to the generation of siRNAs (Waterhouse et al., 1998; Dalmay et al., 2000; Mourrain et al., 2000). Since transitivity is equally dependent on RDR6 function (Vaistij et al., 2002), the same reasons causing transgenes to become self-silenced could also be behind their propensity to support transitivity when targeted by siRNAs. Next, we discuss the different factors that might influence RDR6-dependent silencing of transgenes and the onset of siRNA-triggered transitivity.

#### High Levels of Transgene Expression

One of the first factors used to explain the higher susceptibility of transgenes to the RDR6 pathway is the high levels of expression usually associated to those lines. Often, transgene expression is driven by strong, constitutive promoters, such as the cauliflower mosaic virus (CaMV) 35S regulatory sequence. Indeed, several reports showing transitivity in plants involved the expression of a transgene using the 35S promoter (Braunstein et al., 2002; Vaistij et al., 2002; Himber et al., 2003; Kościańska et al., 2005; Miki et al., 2005; Petersen and Albrechtsen, 2005; Bleys et al., 2006a; Bleys et al., 2006b; Dadami et al., 2013). In petunia, silencing of the endogenous *CHALCONE SYNTHASE* (*CHS*) gene by co-suppression was positively correlated with the strength of the 5′ regulatory sequence used, with silencing efficiency increasing when stronger promoters were employed (Que et al., 1997). In addition, the degree of co-suppression detected for *CHS* was positively related to the transgene copy number, suggesting a dosage effect (Jorgensen et al., 1996). Analysis of a population of transgenic plants has shown that higher transgene expression can correlate with higher numbers of insertions. However, over a certain copy number, silencing is likely to be triggered, suggesting that excessively transcribed genes (Schubert et al., 2004), or the likelihood of insertion as an inverted repeat (Waterhouse et al., 1998) gives rise to sRNAs. Possibly, the high levels of expression linked to the transgene could be recognized by the plant cell as a sign of non-self, triggering a defense mechanism dependent on siRNAs. Alternatively, the intense transcriptional activity supported by such strong promoters could result in an increased number of aberrant mRNAs being generated. Accordingly, transcripts lacking a poly(A) tail have been shown to be substrates for RDR6 activity (Luo and Chen, 2007; Baeg et al., 2017). Nevertheless, several lines of evidence refute such a scenario. As discussed before, miRNA targets, many of which highly expressed, usually do not sustain transitivity, except for events involving programmed AGOs (de Felippes, 2019). Despite being the most expressed gene in plants, transitivity was never detected when the endogenous RuBisCO was targeted by siRNAs (Vaistij et al., 2002; Himber et al., 2003). Furthermore, even when expression of the endogene *VIRP1* is controlled by the CaMV 35S promoter, siRNAs targeting this locus do not trigger transitivity, contrasting with a GFP reporter driven by the same regulatory sequence (Kościańska et al., 2005). Nonetheless, the level of transcript that can serve as template for RDR6 seems to affect the level of secondary siRNAs produced, and therefore, the efficiency of the silencing signal (García-Pérez et al., 2004; Bleys et al., 2006a).

#### Structure and Sequence of the Transgene-Derived Transcript

Most transgenic constructs designed for plant transformation consist of a cDNA regulated by a promoter and terminator of viral or bacterial origin, such as the CaMV 35S, the *OCTOPINE SYNTHASE* (*OCS*) and the *NOPALINE SYNTHASE* (*NOS*) regulatory sequences. This specific configuration is strongly related to prokaryotic genes, and could thus, be recognized by plants as a foreign nucleic acid that needs to be shut down. The idea that such features in a transgenic sequence are perceived by the plant cell and differently handled when compared to an endogenous gene is supported by studies using a GFP reporter construct designed to mimic an endogene (Dadami et al., 2013; Dadami et al., 2014). In these studies, GFP was expressed as a genomic sequence carrying the introns and the regulatory regions from the tobacco RuBisco small subunit gene. Compared to a classical GFP construct containing the reporter cDNA under the 35S promoter and the *NOS* terminator regulation, the endogene-resembling GFP was more stably expressed, supported less siRNA production and showed delayed onset of local and systemic silencing (Dadami et al., 2013). Most interestingly, no transitivity could be detected in plants stably expressing the modified reporter after a hpRNA targeting GFP was used to trigger RNA silencing (Dadami et al., 2014).

To simplify transgenic constructs, they often incorporate cDNA rather than intron-bearing genomic sequences. Thus, plants could use this feature to differentiate between self and non-self sequences and make transcripts that undergo splicing more invisible to the RDR6 pathway. Indeed, genome-wide analysis in Arabidopsis revealed a positive correlation between intronless loci and the presence of endogenous sRNAs (Christie et al., 2011). In addition to the example presented previously, where a GFP mimicking the RuBisCo gene does not show signs of transitivity, other lines of evidence corroborate the idea that transcripts carrying introns are more stably expressed and less susceptible to become templates of RDR6. For instance, transient expression efficiency of GFP using viral replicons is dramatically improved when multiple introns are added to the reporter sequence (Marillonnet et al., 2005). A similar effect was also observed when an intron-containing GFP transgene was agro-infiltrated in *Nicotiana benthamiana* leaves, although this effect was dependent on the accompanying terminator sequence (de Felippes et al., 2020). Furthermore, the abundance of RDR6-dependent siRNAs originating from a 35S-driven transgene is reduced when a GFP containing introns is used (Christie et al., 2011). Transitivity, however, is still detected in transgenes carrying introns, but the onset, frequency, and efficiency are decreased in such constructs, with the reduction being proportional to the number of introns that were added (Vermeersch et al., 2010). However, this effect of introns on transitivity is most likely related to the addition of a sequence between the different elements of the reporter transgene and a possible limitation in the range of RDR6 activity, which will be discussed in more detail later on. Nonetheless, the effect that introns have in repressing silencing suggests a competition between this mechanism and splicing. So, in the presence of introns, transcripts would be favorably routed away from RDR6 and the silencing pathway. The fact that in *C. elegans* RNA silencing of endogenous genes, but not transgenes, requires the action of an RNA helicase supports the notion that introns add an extra layer of protection to these loci (Akay et al., 2017).

#### Efficiency of Transgene Transcriptional Termination

Usually relegated to a feature of less significance, it is becoming clear that terminators and transcriptional termination are important elements for a strong and stable expression of transgenes. During transcription, sequences in the terminator region are recognized by the 3′ end processing machinery resulting in the addition of a poly(A) tail to the nascent transcript and triggering the end of the polymerization process by the RNA polymerase II (Mandel et al., 2007; Kumar et al., 2019; Thore and Fribourg, 2019). A growing number of studies have shown that different terminators can have a distinct influence in the efficiency of transgene expression (Ingelbrecht et al., 1989; Nagaya et al., 2009; Yang et al., 2009; Hirai et al., 2011; Pérez-González and Caro, 2018; de Felippes et al., 2020). The improper formation of the mRNA 3′ end has also been implicated with enhanced silencing of transgenes. Several members of the polyadenylation machinery have been identified in mutagenesis screens where silencing of GFP was used as a reporter system (Herr et al., 2006; de Felippes et al., 2020). Plants carrying mutations in those genes presented increased levels of read-through, which could lead to transcripts missing a poly(A) tail. In accordance, Luo and Chen (Luo and Chen, 2007) observed that improperly terminated transcripts lacking a poly(A) tail are target by RDR6 and become template for dsRNA production. Likewise, the existence of the poly(A) tail seems to inhibit the initiation of dsRNA synthesis by RDR6 (Baeg et al., 2017). These studies indicate that an efficient termination of transcription is a key step to protect genes from become silenced. Indeed, genes lacking a transcriptional termination signal are potent inducers of RDR6-dependent secondary siRNA production, while the use of double terminators can improve 3′ end processing and protect transgenes from silencing (Luo and Chen, 2007; Nicholson and Srivastava, 2009; Yamamoto et al., 2018). The key function of terminators in avoiding mRNAs being channeled to RDR6 has been made evident in a recent study analyzing the role of different genetic elements in the onset of transgene silencing. Compared to promoters and the presence of introns, terminator usage was the factor that most impacted the production of sRNAs originating from a GFP reporter transgene (de Felippes et al., 2020). Interestingly, the protective role of terminators could be seen even when strong promoters were used, suggesting that adverse effects caused by high levels of transgene expression, as the one induced by the CaMV 35S, could be counter-balanced by strong 3′ regulatory sequences. Thus, it is possible that endogenes had evolved to have compatible regulatory sequences, resulting in low levels of aberrant transcripts being produced, and consequently, avoiding the mRNA being routed to the RDR6 pathway. Transgenic constructs, in contrast, could carry insufficiently strong terminators when used with most of the popular strong promoters.

#### Competition With the RNA Decay Pathway

Eukaryotic cells need to keep an appropriated balance of their mRNAs and quickly eliminate aberrant transcripts that might interfere with the proper cell function. An important mechanism to achieve this is through the RNA decay pathway, which degrades mRNAs missing their poly(A) tail or the 5′ cap. In plants, uncapped transcripts are efficiently degraded by XRN4, while mRNAs missing the poly(A) became targeted by the RNA exosome, which is a multimeric complex that includes SKI2, SKI3 and SKI8, among others (Souret et al., 2004; Moreno et al., 2013; Branscheid et al., 2015; Yu et al., 2015; Zhang et al., 2015). The RNA decay pathway is also responsible for eliminating transcripts cleaved by sRNAs (Souret et al., 2004). Interestingly, suppression of this degradation mechanism results in increased levels of sRNAs originating from endogenous genes, including miRNA targets (Gregory et al., 2008; Branscheid et al., 2015; Yu et al., 2015; Zhang et al., 2015). Transgene expression is also affected by the lack of XRN4 and the exosome functions. In plants carrying mutations in these factors, transgenes become silenced, and in both cases, the appearance of siRNAs is dependent on RDR6 (Gazzani et al., 2004; Moreno et al., 2013; Branscheid et al., 2015; Yu et al., 2015; Zhang et al., 2015). Plants also code for two nuclear exoribonucleases, XRN2 and XRN3. In mutants where the expression of these factors is compromised, transgene silencing was also enhanced, however, in much lower levels than has been described for the cytoplasmic XRN4 (Gy et al., 2007). In order to be targeted for XRN4/exosome degradation, some mRNAs need to go through a process of decapping or deadenylation of the poly(A) tail (Liu and Chen, 2016). In plants carrying mutations in factors involved in these preliminary steps, several endogenes become sources of siRNA, and S-PTGS of transgenes is boosted (Thran et al., 2012; Moreno et al., 2013; Martínez de Alba et al., 2015). Thus, a competition between the RNA decay and the RDR6 pathway seems to exist for access to aberrant transcripts. In the case of most endogenes and heritably stable transgenes, the RNA decay mechanism would be dominant, leading to efficient degradation of aberrant transcripts and sRNA targets. In contrast, the transgenic mRNAs in unstable lines might escape the exosome and XRN4, thus retaining aberrant transcripts to feed the RDR6 pathway. This would explain their bias toward transitivity.

What are the signals responsible for sorting mRNAs into one or the other pathway? The answer could lie in some of the aspects discussed previously, such as improperly terminated transcripts, possible structures present in genetic elements usually utilized for transgene expression, or the lack of them, as in the case of transcripts without introns. *HEN2* (*HUA enhancer 2*) is another gene that when mutated leads to increased S-PTGS (Lange et al., 2014). The product of this gene interacts with several components of the exosome and in *hen2* plants several transcripts known to be substrates of this degradation pathway accumulate. Among these transcripts, many genes that might go through alternative 3′ end processing or read-through can be found, as well as incompletely spliced molecules. In addition, HEN2 seems to interact with proteins of the exon junction complex, further implicating splicing as a possible feature used by the cell to sort transcripts between RNA decay or PTGS (Lange et al., 2014). Moreover, the possible role of splicing in this sorting process is supported by the finding that SERRATE (SE) and the cap binding protein ABH1/CBP80 are required for the intron-mediated suppression of transgene silencing (Christie et al., 2011; Christie and Carroll, 2014); and that *se* and *abh1/cbp80* accumulate several transcripts showing splicing defects (Laubinger et al., 2008). Finally, many alternative splicing variants and transcripts with long 3′ UTR are tagged by the nonsense-mediated mRNA decay (NMD) machinery, a RNA surveillance mechanism that recognizes aberrant mRNAs and sends them to the exosome/XRN4 for degradation (Kertész et al., 2006; Ohtani and Wachter, 2019). Mutations in factors of this pathway have also been shown to increase PTGS of a transgene reporter (Moreno et al., 2013).

## Transitivity Spreading Pattern in Plants

Transitivity is not exclusive to plants, but is also present in other organisms that have RNA-dependent polymerases, such as nematodes and fungi (Sijen et al., 2001; Nicolás et al., 2003). In *Caenorhabditis elegans*, transitivity is only detected in the 5′ region of the original targeted site (Sijen et al., 2001; Alder et al., 2003). In fungi, secondary siRNA has been reported to originate from either region, with transitivity occurring upstream for *Schizosaccharomyces pombe* and *Aspergillus oryzae* (Simmer et al., 2010; Fernandez et al., 2012), or downstream of the targeted area for *Mucor circinelloides* (Nicolás et al., 2003). In plants, transitivity is bi-directional, with the silencing signal spreading to both directions (Vaistij et al., 2002). Yet, the expansion of the silencing signal in plants shows a clear tendency toward the 3′ region of the transcript (Braunstein et al., 2002; Petersen and Albrechtsen, 2005; Bleys et al., 2006b; Haque et al., 2007; Moissiard et al., 2007; Aregger et al., 2012; Dadami et al., 2014; Wroblewski et al., 2014; Han et al., 2015; de Felippes et al., 2020). How might this be explained?

**Figure 2:**
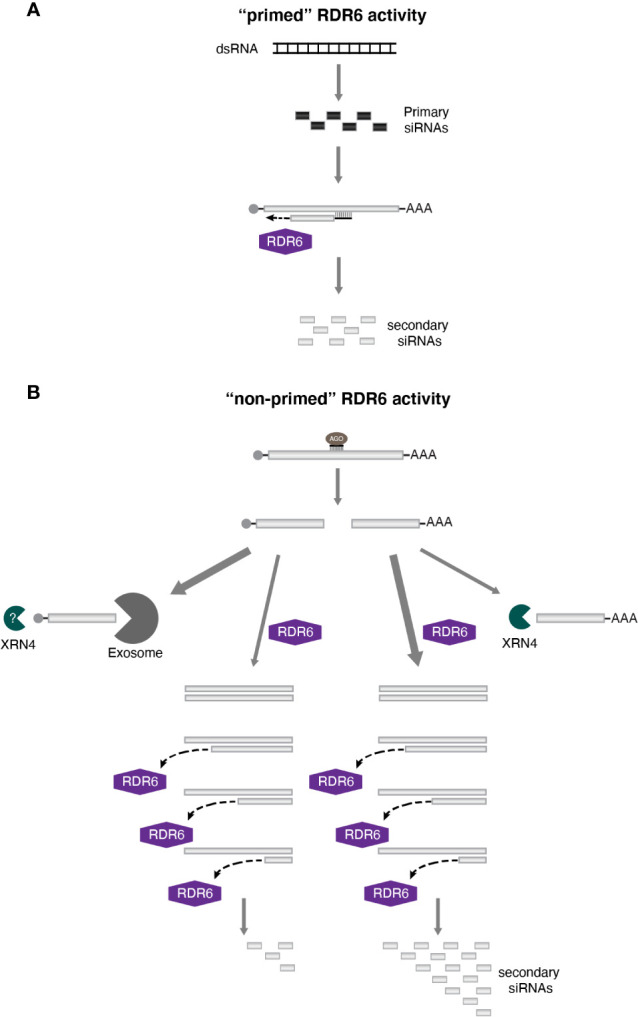
The sequence-spread of transitivity in plants. Transitivity can spread bi-directionally in plants. **(A)** One possible mechanism to explain 3′-5′ silencing spreading involves the use of the primary siRNA as a primer for RDR6 activity. **(B)** However, given that secondary siRNAs are often more abundant at the 3′ end of the target mRNA, “non-primed” RDR6 function would be the most likely mechanism accounting for the pattern of transitivity spreading detected in plants. dsRNA synthesis by RDR6 would start at the 3′ end of the template sequence, and it would be impacted by other factors such as the more effective degradation of the 5′ cleavage fragment (indicated by the width of the arrow), and a drop in efficiency of RDR6 during transcription (illustrated by the disassociation of RDR6 from its template before reaching the end of the molecule). Taken together, all this factors would justify the transitive signal being strongly detected at the 3′ end of the target transcripts.

A hallmark of polymerases is the extension of the nascent strand with new nucleotides being added following a 5′-3′ direction. The spread of transitivity to regions upstream of the target sequence is consistent with this directionality of DNA and RNA polymerases, since elongation of the complementary strand would occur toward the 5′ end of the transcript (**Figure 2A**). In addition, the sRNA triggering secondary siRNA production could act as a primer, setting the location where cDNA synthesis would start and providing the 3′ end OH required for the activity of some polymerases. Indeed, this is the current model to explain the spread of silencing through transitivity in *C. elegans* (Sijen et al., 2001). It is possible that this same mechanism also accounts for the spreading of transitivity to the 5′ region in plants (3′-5′ direction). However, the high level of secondary siRNAs accumulating from the 3′ region of the target transcript suggests an alternative mechanism.

Several authors have proposed that, in order for the 5′-3′ spread of transitivity to occur, RDR6 activity would most likely involve an unprimed synthesis of the complementary strand (Vaistij et al., 2002; Tang et al., 2003; Petersen and Albrechtsen, 2005; Bleys et al., 2006b). Supporting this view, early characterization of a tomato RDR has shown that this enzyme is capable of generating dsRNA with or without a primer (Schiebel et al., 1993). Similar conclusions were made by Tang and colleagues (Tang et al., 2003) when using wheat germ extract. The authors showed that a plant extract contains RDR activity able to convert single strand RNA (ssRNA) into dsRNA, and consequently siRNAs, without the presence of primers. In addition, only unprimed activity was detected *in vitro* for the Arabidopsis RDR6 (Curaba and Chen, 2008). More recently, the primer-dependent activity of RDR6 was also demonstrated *in vitro*, but this was less efficient than the non-primed functionality of this enzyme (Devert et al., 2015). Interestingly, in all these works, the unprimed activity of RDRs tend to initiate at or close to the 3′ end of the template molecule. This observation is corroborated by the analysis of tasiRNA biogenesis. Both, *TAS1* and *TAS2* transcripts are converted to dsRNAs after being targeted by miR173, resulting in the generation of tasiRNAs. Analysis of the dsRNA precursor revealed that synthesis of the complementary strand encompassed the whole transcript, included the poly(A) tail (Rajeswaran et al., 2012). Likewise, RDR6-dependent dsRNA synthesis having as template *TAS3* transcripts, which are targeted by miR390, starts from the third most terminal nucleotide of the fragment spawning tasiRNAs (Rajeswaran and Pooggin, 2012). Therefore, a scenario arises where transitivity in plants relies mostly on a non-primed activity of RDR6, with dsRNA synthesis initiating preferentially at the 3′ end of the template sequence. This modus operandi would ensure that this region is frequently converted into dsRNA, contributing for the over-representation of transitive siRNAs detected in the 3′ region of a transcript (**Figure 2B**).

Nonetheless, other factors need to be taken in consideration and probably contribute for the preferred spreading of transitivity toward the 3′ region of the target transcript. With RDR6 transcription initiating at the 3′ extremity of a sequence, siRNA production from parts of the transcript located closer to the 5′ end of the RNA would rely on the complementary strand synthesis continuing until it reaches such regions. Different studies have estimated the range of RDR6 processivity and indicate that the enzyme could support RNA synthesis spanning at least 750 nt (Petersen and Albrechtsen, 2005; Bleys et al., 2006b; Moissiard et al., 2007; Vermeersch et al., 2010). However, RDR6 efficiency seems to decrease the further a region is from the 3′ termini of the RNA. In Arabidopsis, the accumulation of transitive siRNAs originating from a reporter gene was shown to decline progressively toward the 5′ end of the transcript (Moissiard et al., 2007). The drop in RDR6 processivity is also supported by studies involving the “XYZ” reporter system. In this system, plants express three different constructs: the “X” part codes for a trigger of RNA silencing, such as a hairpin RNA, targeting the 3′ region of “Y,” while “Z” carries a reporter gene that share sequence similarities with the 5′ end of “Y.” Since there is no overlap between the region targeted by “X” and the gene in “Z,” silencing of the reporter can only be detected when transitivity on the “Y” construct is occurring. It has been reported that the efficiency and frequency of transitivity-dependent silencing on the reporter gene can be negatively affected when the space between the 3′ region and the “Z” overlapping sequence in “Y” is increased (Bleys et al., 2006b; Vermeersch et al., 2010). Possibly, a significant fraction of RDR6-dependent RNA synthesis would not reach regions that are too far from the transcription beginning, even if the sequence is within range of the enzyme activity, due to early disassociation of the polymerase from the template (**Figure 2B**). All together, these factors would further contribute for the preferential spread of transitivity observed in direction of the 3′ region of transcripts.

The consequences of the primary sRNA targeting event also need to be taken into consideration. After cleavage of the target RNA, two fragments will be formed (a 5′ and a 3′ one). In principle, this should not contribute for the over-representation of siRNAs being produced from the 3′ region, since both cleavage fragments are produced at the same ratio, and the 5′ one could also support RDR6 activity in ways similar to the one seen for the 3′. But this seems not to be the case. As discussed before, each of these fragments is degraded by different processes. The 5′ fragment, which no longer carries a poly(A) tail, is degraded by the exosome, while the uncapped 3′ fragment is targeted by XRN4, with both processes competing with RDR6 for the target transcript (Gazzani et al., 2004; Souret et al., 2004; Gregory et al., 2008; Moreno et al., 2013; Branscheid et al., 2015; Yu et al., 2015; Zhang et al., 2015). Therefore, any difference in efficiency between this two degradation processes could affect the amount of complementary RNA molecules synthesized by RDR6, since the quantity of template seems to have an impact on the levels of transitive silencing (Bleys et al., 2006a). Comparison between *ski3* (one of the components of the exosome) and *xrn4* mutants revealed that the former has a weaker effect on the onset of S-PTGS than the latter (Yu et al., 2015). The authors suggested that when 3′ to 5′ degradation is compromised, decapping of the 5′ fragment would occur, exposing the transcript to XRN4 activity and limiting the exposure to the RDR6 pathway. As a consequence, most siRNAs would be produced from a dsRNA that would originate from the 3′ cleavage fragment, containing sequences from the 3′ end of the original transcript up to the cleavage site, which would once more, contribute to a stronger level of transitivity in the 5′-3′ direction (**Figure 2B**).

## Final Remarks

Despite the importance of transitivity to plant defense and development, several aspects of the mechanism are still poorly understood. In this review, we focused on two characteristics that are unique to this process: the considerable bias toward the production of secondary siRNAs from transgenic transcripts, and the preferential spreading of the silencing signal to the 3′ end of the gene.

Several factors have been identified and used to explain the susceptibility of transgenes to transitivity, especially when triggered by siRNAs. Among them, the proper formation of the mRNA 3′ end seems to be of major importance, directly influencing the recruitment of RDR6 and compensating for the negative effect that might be associated with other elements, such as the high expression levels of some transgenes and the origin of their regulatory sequences. The competition between the RNA decay pathway and the silencing pathway also seems to play a major role, and that defects introduced into the RNA degradation machinery strongly induce PTGS. However, the biological relevance of this interplay still needs to be tested. Given the universal role of the exosome and XRN4 in degrading aberrant mRNAs, it seems unlikely that changing the efficiency of this process would be a reliable way for the cell to control gene expression, since it would probably affect several unrelated genes. Nonetheless, the identification of cell types or physiological conditions where the activity of the RNA decay pathway is altered would be a good indication that cells do indeed modulate the degradation of mRNAs to control gene expression.

Despite all the advances in this field, some aspects of siRNA-triggered transitivity are still elusive. For instance, while many features affecting the onset of transitivity occur in the nucleus, such as splicing and transcription termination, RDR6 and other factors involved with secondary siRNA generation are mainly present at the cytoplasm, most specifically at specialized foci known as siRNA bodies (Glick et al., 2008; Elmayan et al., 2009; Kumakura et al., 2009; Jouannet et al., 2012; Pumplin et al., 2016). This difference in localization implies that unknown factors exist that mediate the cross-talk between these cellular structures and allow potential RDR6 templates to be transported to such RDR6-containing structures. Another aspect requiring further investigation refers to the mechanisms and factors recognizing aberrant transcripts, such as mRNAs missing the poly(A) tail. Concerning the transitivity triggered by miRNAs, investigating how 22 nt/asymmetric molecules can re-program AGOs to support secondary siRNA production, and what makes AGO7 able to do the same, would be of extreme value to better understand the biology of phasiRNAs.

There is no doubt that transitivity plays a central role in many different processes in plants. It adds more plasticity to the gene regulation mediated by sRNAs and at the same time boosts the intensity of the silencing signal. However, this process needs to be strictly regulated to avoid sRNA silencing going out of control and spreading to loci that should not be downregulated. The onset of transitivity has most likely evolved to be a rare event, occurring only in certain conditions. This is probably reflected in the dominance of DCL4 over DCL2 and in the relative low abundance of 22 nt sRNAs, which are known to be triggers of secondary siRNA biogenesis. The fact that transgenes are more sensitive to transitivity might be a consequence of the participation of this process in the defense against exogenous sequences, and therefore, part of the mechanism allowing plants to recognize “self” from “non-self.”

## Author Contributions

FFF and PMW conceived, performed literature review, and wrote the manuscript.

## Conflict of Interest

The authors declare that the research was conducted in the absence of any commercial or financial relationships that could be construed as a potential conflict of interest.
